# Nasal vaccination against *Trypanosoma cruzi*: a dual approach for prevention and treatment of chronic Chagas cardiomyopathy

**DOI:** 10.3389/fimmu.2025.1662036

**Published:** 2025-10-10

**Authors:** Paula Cacik, Maria Florencia Pacini, Camila Bulfoni Balbi, Brenda Dinatale, Genaro Diaz, Ulises Cha, Cecilia Farré, Mónica Gianeselli, Estefanía Prochetto, Ana Rosa Peréz, Iván Sergio Marcipar

**Affiliations:** ^1^ Laboratorio de Tecnología Inmunológica Facultad de Bioquímica y Ciencias Biológicas- Universidad Nacional del Litoral-Consejo Nacional de Investigaciones Científicas y Técnicas (LTI-FBCB- CONICET), Santa Fe, Argentina; ^2^ Instituto de Inmunología Clínica y Experimental de Rosario (IDICER-CONICET-UNR) and CIPREB, Facultad de Cs. Médicas, Universidad Nacional de Rosario, Rosario, Argentina; ^3^ Facultad de Ciencias Veterinarias, Universidad Nacional del Nordeste, Corrientes, Argentina

**Keywords:** vaccine, *Trypanosoma cruzi*, chronic infection, therapeutic, prophilactic

## Abstract

Chagas disease, caused by the parasite *Trypanosoma cruzi* (*T. cruzi*), is a neglected life-threatening disease. Given that available pharmacologic treatments are effective only in the acute phase, and that diagnosis typically occurs during the chronic phase when cardiac damage is already present, current efforts should aim to mitigate cardiac pathology during chronic infection. This study evaluates the effectiveness of a nasal vaccine based on trans-sialidase (TS) plus c-di-AMP in both prophylactic and therapeutic settings against chronic Chagas cardiomyopathy (CCC) in a mouse model of *T. cruzi* oral infection. Prophylactic and therapeutic vaccination significantly reduced cardiac inflammation, fibrosis, and parasite load. Histological analysis confirmed less cardiac damage in vaccinated groups compared to infected, unvaccinated controls. While electrocardiographic abnormalities were fully prevented in the prophylactic group, therapeutic vaccination still halved arrhythmia incidence, indicating functional benefits despite late administration. Immunologically, both vaccine regimens promoted a Th17-skewed response, with increased IL-17 expression in cardiac tissue. However, distinct immune signatures were observed: prophylactic vaccination reduced TGF-β and T-bet expression, correlating with less fibrosis and inflammation; therapeutic vaccination elevated Foxp3, suggesting regulatory T cell involvement in controlling chronic inflammation. Both strategies enhanced TS-specific antibodies and reduced non-protective, parasite-wide antibody responses, shifting the humoral profile toward functional protection. Importantly, vaccinated animals also showed a marked reduction in heart auto-reactive antibodies. The findings suggest that early intervention yields greater benefits, but even post-infection, immunization can also significantly mitigate cardiac damage. These results underscore the potential of nasal TS-based vaccines as a non-invasive, dual-action strategy to both prevent and treat CCC.

## Introduction

Chagas disease is endemic in Latin America, where it affects more than 6–7 million people; however, in recent years, it has been increasingly reported in other regions due to migration (1). The disease is caused by the parasite *Trypanosoma cruzi*, which produces nonspecific symptoms during the acute phase of infection, typically resolving within 1 to 2 months. Subsequently, the infection enters an asymptomatic phase, which may persist for a lifetime. However, in 30-40% of cases, patients develop cardiac or intestinal lesions that can be debilitating and life-threatening (2). The currently available treatments, Benznidazole (Bz) and Nifurtimox (NFX), have a limited efficacy during the chronic phase of infection (3). Therefore, minimizing the development of lesions during the chronic phase of *T. cruzi* infection remains a major goal for controlling Chagas disease.

Although most vaccines are designed to avoid infections or lessen the severity of symptoms during the acute phase, they can also help to prevent long-term damage caused by chronic or latent infections (4). Unlike drug treatments, therapeutic vaccines do not act directly on the disease; instead, they stimulate the immune system to combat the infection. This approach, currently explored for chronic infections such as hepatitis B, human papillomavirus, or Epstein-Barr virus, may eliminate the pathogen and its associated disease cause (4). However, even when sterilizing immunity is not achieved, therapeutic vaccines can still help alleviate disease symptoms by modulating immune response patterns and promoting a new balance between the pathogen and the host (5). In the context of *T. cruzi* infection, where complete parasite clearance is challenging and most diagnoses occur during the chronic stage—when antiparasitic drugs are no longer effective—such immune-based strategies represent a particularly valuable alternative.

In our group, we have developed a nasal vaccine based on the hypothesis that it could provide protection not only against infections transmitted through vector bites or transfusion, but also via contaminated food. This approach relies on the premise that a nasal vaccine targeting mucosal immunity at key entry points—such as the conjunctiva and buccal cavity—can effectively stimulate both systemic and mucosal immune responses. This is particularly relevant given the rise in orally transmitted *T. cruzi* cases over the past two decades (1), which has led to Chagas disease being recognized by the WHO as a relevant food-borne disease in Latin America. The increase in orally transmitted cases, often associated with severe symptoms and higher mortality rates due to acute cardiomyopathy, underscores the urgent need for better strategies to manage oral Chagas disease. As reported in our previous studies, our nasal vaccine candidate, formulated with Trans-sialidase (TS) and cyclic di-adenosine monophosphate (c-di-AMP), has demonstrated protective effects against acute oral *T. cruzi* infection (6, 7).

The TS surface family of proteins in *T. cruzi* comprises eight groups, with Group-1 exhibiting enzymatic trans-sialidase activity—a well-known virulence factor of the parasite (7, 8). This protein has been shown to provide significant protection when used in experimental vaccines across various platforms (9). Recently, we reported that TS contains conserved T and B cell epitopes, supporting its potential as a promising vaccine candidate (7). Moreover, we have confirmed its immunogenic and/or protective capacity in several studies, using different formulations and administration routes (6, 10–15).

Particularly, for the development of a mucosal vaccine, we designed a formulation using a recombinant fragment of the Group-1 TS protein, combined with c-di-AMP as an adjuvant. The nucleotide c-di-AMP, a bacterial second messenger (16) acts as a potent immunomodulatory adjuvant in mice, and shows promise for use in humans. The c-di-AMP activates the STimulator of INterferon Genes (STING) pathway, leading to the release of type I interferons (IFNs) and other proinflammatory cytokines (17). In addition, intranasal administration of c-di-AMP has previously been shown to promote a balanced Th1/Th2 immune response (18).

Recently, we reported in a preclinical setting that nasal administration of the vaccine formulation combining a recombinant TS fragment with c-di-AMP (TS+A), attenuated both clinical manifestations and cardiac lesions during the acute phase of oral *T. cruzi* infection. The protection conferred by TS+A extended into the chronic phase, resulting in a clear reduction in chronic myocarditis, fibrosis, and functional electrocardiographic abnormalities. This effect was associated with decreased expression of the profibrotic cytokine Transforming Growth Factor Beta (TGF-β) (13). In addition, the TS+A formulation elicited a robust response at the nasal-associated lymphoid tissue (NALT), influencing both systemic humoral and cellular immune responses, and significantly reducing parasitemia and parasite load in the heart, skeletal muscles, and intestines, along with markers of hepatic and muscular damage. These results demonstrate the feasibility of developing a mucosal vaccine against *T. cruzi* based on TS and c-di-AMP, capable of mitigating the progression of chronic Chagas cardiomyopathy (CCC) (13).

Given the evidence supporting the beneficial effect of TS+A prophylactic vaccination upon the chronic phase of infection—and recognizing that most Chagas disease patients are diagnosed at this later stage—we also aimed to evaluate its potential as a therapeutic tool. To this end, we compared the degree of protection against chronic disease progression as well as the associated immune responses elicited by prophylactic versus therapeutic vaccination strategies.

## Methods

### Nasal vaccination schedules

BALB/c female mice aged 6–8 weeks at the beginning of the experimental schedules were used. To establish prophylactic and therapeutic schedules, mice were immunized intranasally, either before or after infection, respectively. Formulations containing 10 µg of N-terminal TS fragment and 5 µg of c-di-AMP were administered in three doses, at two-week intervals, as previously reported (13). All protocols for animal studies were approved by the Institutional Animal Care and Use Committee (Res. No: 6698/2014 and 2958/2018, FBCB-UNL CE2018-42), according to the institutional guidelines and carried out following the National Institutes of Health’s Guide for the Care and Use of Laboratory Animals.

**Figure 1:**
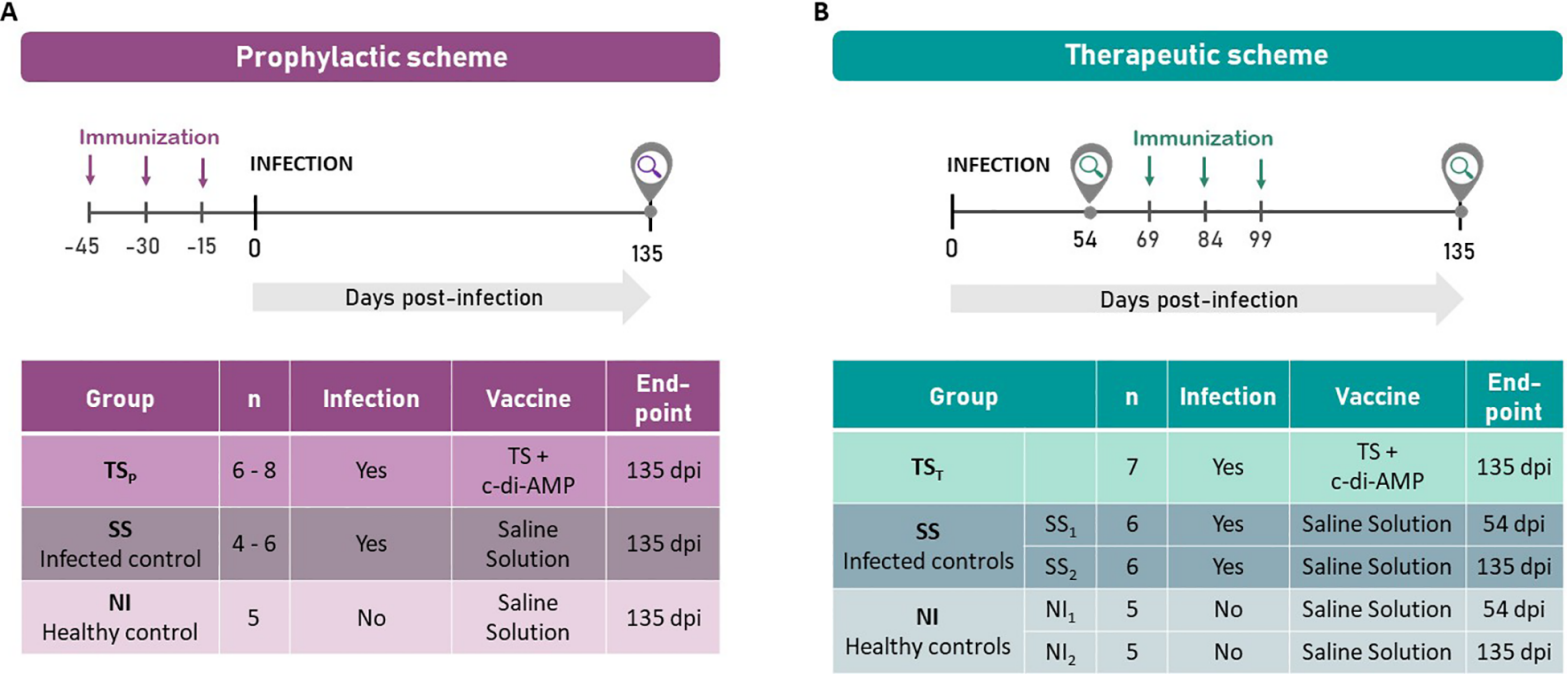
Experimental design for prophylactic **(A)** and therapeutic **(B)** vaccination strategies.

As seen in **Figure 1A**, for the prophylactic approach, mice were divided into three groups (n=4–8/group). One group (TS_P_) received intranasal immunization with TS+A, while the other two groups (SS and NI) were given saline solution via the same route. Fifteen days after completing the immunization schedule, the TS_P_ and SS groups were orally challenged with 3000 bloodstream trypomastigotes of the Tulahuen strain (DTU-VI), whereas the NI group remained uninfected. The experimental end-point was 135 days post-infection (dpi).

For the therapeutic approach (**Figure 1B**), mice were initially divided into five groups (n=5–7/group): TS_T_, SS_1_, and SS_2_ were orally infected with 3000 trypomastigotes, while NI_1_ and NI_2_ remained uninfected. NI_1_ and SS_1_ were sacrificed at 54 dpi for early analysis. After 69 dpi, the TS_T_ and NI_2_ groups were intranasally immunized with TS+A, following the same three-dose schedule used for the TS_P_ group, while SS_2_ group received saline by the same route. The experimental endpoint for TS_T_, NI_2_ and SS_2_ groups was also 135 dpi.

### Histopathology

Hearts were removed at both 54 and 135 dpi, transversely sectioned, fixed in 4% formalin, and embedded in paraffin. Subsequently, 5 µm sections were stained with hematoxylin and eosin (H&E) for the evaluation of inflammatory infiltrates and tissue damage, as previously described (6). Briefly, the intensity of myocarditis inflammation was classified as follows: mild, when a slight infiltration with damage to a few muscle fibers; moderate, when infiltrates compromise 3 to 5 muscle fibers; and severe, when increased infiltrates were observed accompanied with destruction of multiple muscle fibers. Additional heart sections were stained with picrosirius red to assess tissue fibrosis. Fibrosis was classified similarly, according to the size and number of affected cardiac fibers and categorized as mild, moderate, or severe. Based on these criteria, a scoring system was developed for quantification for both inflammation and fibrosis, as follows: normal=0; mild=1; moderate=2 and severe=3. The global score for each group was calculated as the median of values of inflammation or fibrosis obtained from each tissue/animal.

### Electrocardiograms

Cardiac electrophysiological alterations were assessed at 54 and 135 dpi. Mice were anesthetized with a ketamine-xylazine solution (100 mg/kg ketamine and 10 mg/kg xylazine) before evaluation. Electrocardiograms (ECG) were recorded using a mouse-adapted electrocardiograph (CardioCom, Paraná, Argentina) equipped with CC7DerS-Vet 1.0.4 software provided by the manufacturer. This system allowed the measurement of heart rate, QT, and QRS intervals. The QT interval was corrected using the formula described by Mitchell (19) to obtain the corrected QT interval (QTc), adjusting for the natural variation of the QT interval with heart rate-shortening at higher rates and lengthening at lower rates. Leads I and II were analyzed to detect arrhythmic abnormalities, such as tachycardia, bradycardia, premature atrial contractions, and atrioventricular block. The presence of arrhythmias was evaluated by calculating the proportion of mice exhibiting any ECG abnormality within each group.

### Heart parasite load quantification

Hearts were collected from mice at 54 and 135 dpi and transversely sectioned into three parts. One part was used to determine the heart parasite load by quantitative PCR (qPCR) (13). For DNA extraction, the method described previously was followed (20). Briefly, total DNA was extracted after tissue disaggregation in a lysis buffer (10 mM Tris-HCl, pH 7.6; 0.1 M NaCl; 10 mM EDTA; 0.5% SDS; 300 µg/mL proteinase K; Sigma). Samples were incubated at 55 °C for 2 h and subjected to two rounds of extraction with a phenol–chloroform–isoamyl alcohol mixture (25:24:1) (Sigma Chemical Co., USA). DNA was then precipitated by adding cold ethanol (AAPER Alcohol and Chemical Co., Shelbyville, KY, USA) at twice the volume of the sample, followed by incubation at −80 °C overnight. Samples were centrifuged at 13000 rpm for 30 min, washed with 70% ethanol, vacuum-dried, and resuspended in sterile water. DNA concentrations were adjusted to 125 ng/µL for use as templates in qPCR reactions.

The qPCR reactions were performed using HOT FIREPol EvaGreen qPCR Mix Plus (Solis Biodyne) on a StepOne™ Real-Time PCR System (Applied Biosystems). Specific primers were used for the amplification of *T. cruzi* kDNA (S36: 5′-GGT TCG ATT GGG GTT GGT G-3′ and S67rev: 5′-GAA CCC CCC TCC CAA AAC C-3′). A quantification curve for *T. cruzi* was generated following the methodology of Prochetto et al. (21), and a standard curve was constructed for murine DNA quantification, using the mouse-specific primers reported in Cummings and Talenton (20). Parasite load was expressed as equivalent parasites per 250 ng of murine DNA.

### Heart cytokine profiling by RT-qPCR

The cytokine profile was analyzed in the heart at 54 and 135 dpi. Briefly, one-third of a transverse heart section was mechanically homogenized in TRI-Reagent^®^ (Sigma-Aldrich, USA). The RNA was then reverse transcribed into cDNA using RevertAid Reverse Transcriptase (Thermo-Fisher Scientific, USA). Real-time PCR was performed on StepOnePlus equipment (Applied-Biosystems, Waltham, MA, USA) using Mix-5x-HOT-FIREPol^®^EvaGreen^®^ qPCR-Mix Plus with ROX (Solis-BioDyne, Tartu, Estonia). The amplification program included an initial activation step at 95 °C for 15 min, followed by denaturation at 95 °C for 15 s, an annealing temperature between 59-62 °C, and finally an elongation at 72 °C for 20 s, for 40 cycles. Fluorescence was recorded at the end of each extension step, and amplification specificity was verified through melting curve analysis of all final PCR products. Relative gene expression was calculated using the 2^–ΔΔCt^ method (22, 23), with glyceraldehyde-3-phosphate dehydrogenase (GAPDH) used as the endogenous reference gene. Amplification efficiencies were confirmed to be identical or similar between target and reference genes. The primers used are listed in **Table 1**.

**Table 1:** Primers used for cytokine and transcription factor profiles analysis by RT-qPCR.

Gene	Forward (5’-3’)	Reverse (5’-3’)
*IFN-γ*	AGACAATCAGGCCATCAGCAAC	CTCATTGAATGCTTGGCGCTG
*IL-17a*	CAAAGCTCAGCGTGTCCAAA	CTTCCCAGATCACAGAGGGATA
*TGF-β*	TGACGTCACTGGAGTTGTACGG	GGTTCATGTCATGGATGGTGC
*IL-10*	CAAGCCTTATCGGAAATGATCCA	CCTTGTAGACACCTTGGTCTTG
*Foxp3*	AAAGGAGAAGCTGGGAGCTATG	GTGGCTACGATGCAGCAAGAG
*T-bet*	AGCTCACCAACAACAAGGGG	CTGCGTTCTGGTAGGCAGTC
*GAPDH*	GAAGGTCGGTGTGAACGGAT	CGTTGAATTTGCCGTGAGTGGA

### Specific antibody detection in plasma

Blood samples were collected after 135 dpi to evaluate total IgG, IgG1, and IgG2a TS-specific antibody levels. Plasma was obtained by centrifugation at 2000 rpm from heparinized blood and stored at −20 °C until use. ELISA microplates (Nunc-Immuno Maxisorp™, Thermo, Waltham, MA, USA) were coated with 0.5 µg/well of purified recombinant TS or *T. cruzi* Total homogenate (TH), diluted in carbonate–bicarbonate buffer (0.05 M; pH 9.6), and incubated overnight at 4 °C. In addition, cardiac autoreactive antibodies were evaluated by coating the plates with a homogenate of healthy BALB/c mouse heart tissue. Heart tissues were homogenized by mechanical disruption in a RIPA buffer containing protease inhibitor cocktail (Sigma) followed by multiple freeze–thaw cycles to ensure cell lysis and antigen release and then centrifuged at 14,000 x g for 5 min. Supernatant was collected and then transferred into new microfuge tubes. Protein concentrations were determined using the Pierce™ BCA Protein Assay Kit (#A55865), following the manufacturer’s instructions. Absorbance was measured at 560 nm using a BioTek microplate reader.

After incubation, wells were washed three times with PBS-Tween 20 (0.05%) and blocked with PBS containing 5% skim milk for 1 h at 37 °C. Plasma samples were diluted 1:100 in PBS with 1% skim milk and incubated in duplicate in coated wells for 1 h at 37 °C. Specific antibodies against TS, *T. cruzi*, or mouse heart homogenate were detected by incubation with peroxidase-conjugated goat anti-mouse IgG, IgG1, or IgG2a (diluted 1:10000; Abcam) for 1 hour at 37 °C. After washing, TMB substrate ready to use (Invitrogen) was added, and the reaction was stopped with sulfuric acid. Absorbance was measured at 450 nm using an ELISA reader (Bio-Tek Instruments, Winooski, VT, USA).

ELISA cut-off values were calculated as the mean optical density (OD) of the negative control (NI) samples plus two standard deviations. Antibody levels were then expressed as an index representing the ratio between the OD of each sample and the calculated cut-off.

### Statistical analysis

Data were analyzed using non-parametric methods. The Kruskal–Wallis ANOVA test was applied for comparisons involving more than two groups, followed by the Mann–Whitney U test for *post hoc* pairwise comparisons. For comparisons between two independent groups, the Mann–Whitney U test was used. The chi-square test was applied to compare categorical variables. Results are presented as mean ± SEM or as median and range, depending on the distribution of the variable. Statistical analyses were conducted using GraphPad Instat version 4.0 (GraphPad Software, San Diego, CA, USA). Differences were considered statistically significant at *p* < 0.05.

## Results

### Cardiac damage and parasite load in the chronic stage

**Figure 2:**
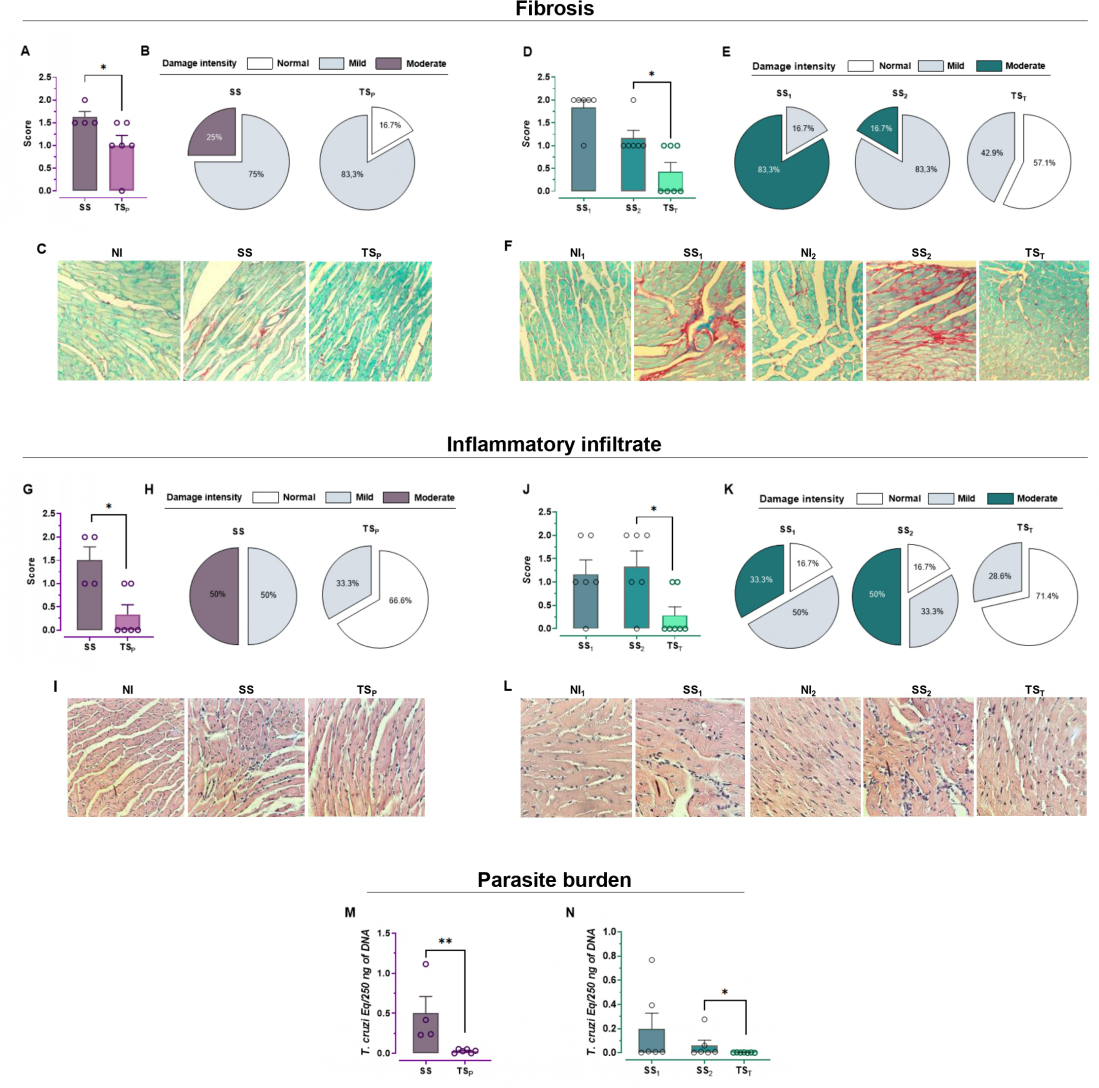
Myocarditis assessment and parasite load: Infiltrates and fibrosis were classified as normal, mild, or moderate. For both the prophylactic (NI = 5, SS = 4, TS_P_=6) and therapeutic (NI_1_ = 5, SS_1_ = 6, NI_2_ = 5, SS_2_ = 6, TS_T_=7) schemes respectively, bar graphs reflect the estimated scores for fibrosis **(A, D)** or tissue damage based on the intensity of inflammatory infiltrates **(G, J)**, while pie charts illustrate the relative proportions of inflammatory infiltrates **(B, E)** and fibrosis in each group **(H, K)**. Representative microscopic images are provided for fibrosis **(C, F)** and for myocarditis **(I, L).** Images were taken at 40× magnification. Parasite burden in cardiac tissue was determined by qPCR for both prophylactic **(M)** and therapeutic **(N)** schedules. Statistical significance is indicated as *p < 0.05 and **p < 0.01.

Inflammatory infiltrate and fibrosis were assessed for all groups and analyzed both quantitatively (**Figures 2A, D, G, J**)—as the mean score values—and qualitatively (**Figures 2B, E, H, K**)—as the proportion of affected mice in each group. Representative images of the stained histological sections are shown in (**Figures 2C, F, I, L**). Among all groups, SS, SS_1_, and SS_2_ exhibited the most pronounced alterations in the evaluated parameters relative to the NI group. Regardless of the vaccination schedule, histological analysis of heart tissue from infected animals revealed statistically significant differences in lesion scores between vaccinated groups (TS_P_ and TS_T_) and their respective unvaccinated controls (SS, SS_1_, and SS_2_), indicating that both treatments reduced the severity of myocarditis (**Figures 2G, J**) and fibrosis (**Figures 2A, D**). This histological protection was further supported by the analysis of the proportion of mice affected by inflammatory infiltrates and fibrosis. In both treatment groups, most mice exhibited normal cardiac tissue, with only a few showing mild infiltrates. In contrast, the majority of mice from the SS groups displayed inflammatory and fibrotic scores ranging from mild to moderate (**Figures 2B, E, H, K**).

Consistent with previous reports for this model (13, 21), the heart parasite load at the chronic stage of unvaccinated, infected groups (SS and SS_2_) was low (with values <0.5 parasite eq/250 ng DNA) (**Figures 2M, N**). Despite the overall low parasite burden, a significant reduction was still observed in mice treated with either the prophylactic (**Figure 2M**) or therapeutic vaccine (**Figure 2N**).

### ECG measurements and arrhythmia analysis

**Figure 3:**
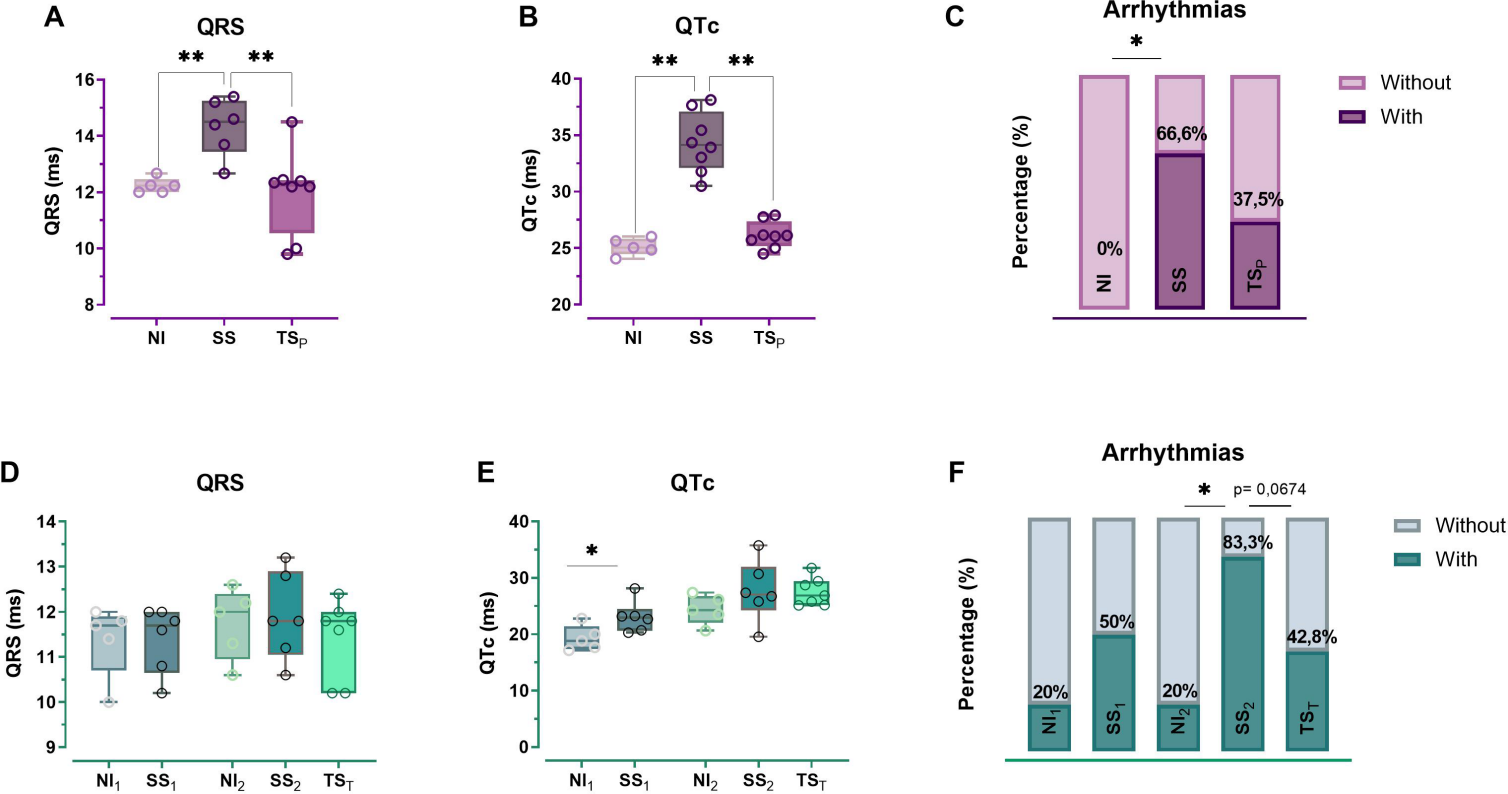
Electrocardiographic alterations: The QRS complex **(A, D)** and corrected QT (QTc) interval **(B, E)** were measured, alongside the detection of arrhythmias **(C, F)** in both prophylactic (NI = 5, SS = 6, TS_P_=8) and therapeutic (NI_1_ = 5, SS_1_ = 6, NI_2_ = 5, SS_2_ = 6, TS_T_=7) schedules, respectively. The levels of significance are defined as follows: *p < 0.05 and **p < 0.01. ms, milliseconds.

QTc and QRS parameters were analyzed quantitatively as the millisecond (ms) mean values, and arrhythmia was analyzed qualitatively as the proportion of affected mice in each group (**Figure 3**). When evaluated the prophylactic schedule at 135 dpi, we confirmed that SS animals showed an increase of QRS intervals and QTc values compared to NI, while TS_P_ group showed a similar behavior in both parameters to NI (**Figures 3A, B**), reinforcing previously reported results (13). However, in the therapeutic schedule, the differences between groups at 135 dpi were not significant for these parameters. In this schedule, only a noticeable prolongation of the QTc interval was observed in SS_1_ vs NI_1_ ECG recordings performed prior to immunization (54 dpi) (**Figures 3D, E**).

In relation to arrhythmias, and as expected, the proportion of affected animals was increased in infected groups at 135 dpi (SS and SS_2_) compared to respective controls (NI and NI_2_). Nevertheless, both treatment schedules appeared to mitigate the increase in arrhythmias during the chronic stage (**Figures 3C, F**). Prophylactic immunization reduced arrhythmia frequency from 66.6% to 37.5% (**Figure 3C**), while therapeutic vaccination lowered it from 83.3% to 42.8% (p = 0.0674; **Figure 3F**), representing an approximate 50% reduction in both cases. Interestingly, in the therapeutic schedule, the SS_1_-infected group, analyzed before the start of immunization, exhibited an arrhythmia incidence of 50%, which increased to 83.3% in the SS_2_-infected group analyzed at 135 dpi. However, this increase was no observed in treated mice (**Figure 3F**).

### Cardiac cytokine and transcription factor expression profiles in the chronic phase after prophylactic or therapeutic vaccination

**Figure 4:**
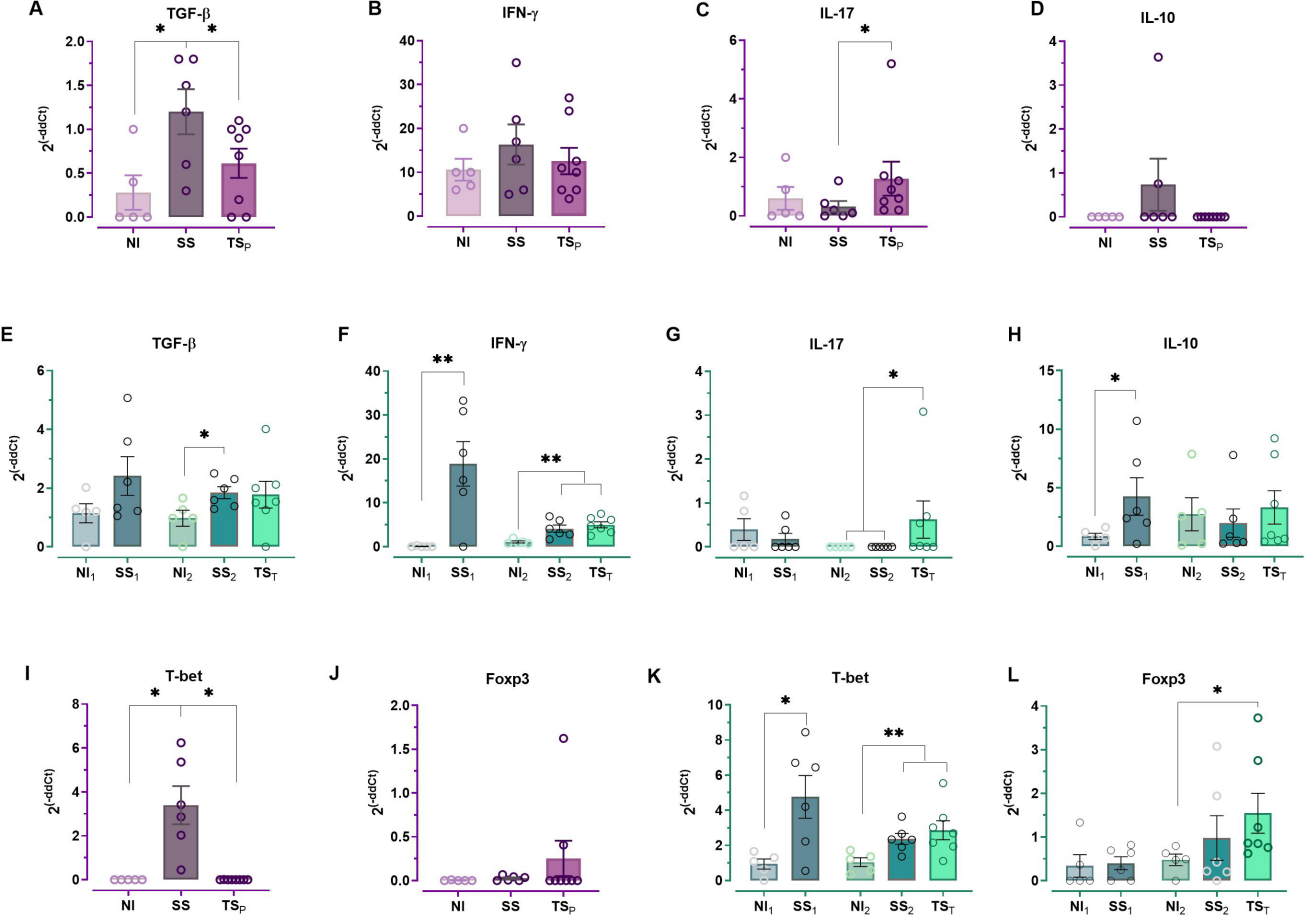
Cytokine and transcription factor profiles in hearts of chronically-infected mice subjects to prophylactic or therapeutic vaccine strategies. Expression profiles were determined by qPCR of mice treated with the prophylactic or therapeutic vaccine, respectively, for TGF-β **(A, E)**; IFN-γ **(B, F)**; IL-17 **(C, G)**; IL-10 **(D, H)**; T-bet **(I, K)**; and Foxp3 **(J, L)**. The levels of significance are defined as follows: *p < 0.05 and **p < 0.01 vs NI. Number of animals per group: prophylactic scheme – NI = 5, SS = 6, TS_P_=8; therapeutic scheme – NI_1_ = 5, SS_1_ = 6, NI_2_ = 5, SS_2_ = 6, TS_T_=7.

As seen in **Figure 4**, analysis of cytokine gene expression in cardiac tissue from chronically infected groups under both schedules (SS and SS_2_) revealed an upregulation of TGF-β compared to their respective non-infected controls (NI and NI_2_), while IL-17 and IL-10 levels remained unchanged. Both SS and SS_2_ groups exhibited elevated IFN-γ levels, although statistical significance was achieved only in the SS_2_ group. Infection also led to an increase in T-bet expression, in line with IFN-γ up-regulation. No increase in Foxp3 expression was observed in both SS and SS_2_ animals.

Prophylactic vaccination significantly reduced TGF-β expression in TS_P_ mice to levels comparable to NI mice, while IL-17 was markedly elevated relative to both NI and SS groups. No significant changes were observed in IFN-γ or IL-10. T-bet expression was significantly reduced in TS_P_ mice compared to SS, whereas Foxp3 levels remained unchanged.

Therapeutic vaccination also led to significant increases in IL-17 but in contrast to Prophylactic vaccination, it led to Foxp3 expression in TS_T_ mice compared to SS_2_. In TS_T_ mice, TGF-β and IFN-γ levels remained similar to those in SS_2_ mice, as did T-bet expression, which differed from the pattern seen in the prophylactic group. IL-10 levels were similar across all groups, despite the increase in Foxp3.

Altogether, these profiles indicate a shift toward a Th17-skewed response in both immunization strategies, with distinct cytokine and transcription factor signatures depending on the timing of vaccination.

### Chronic-phase antibody response following prophylactic or therapeutic vaccine administration

**Figure 5:**
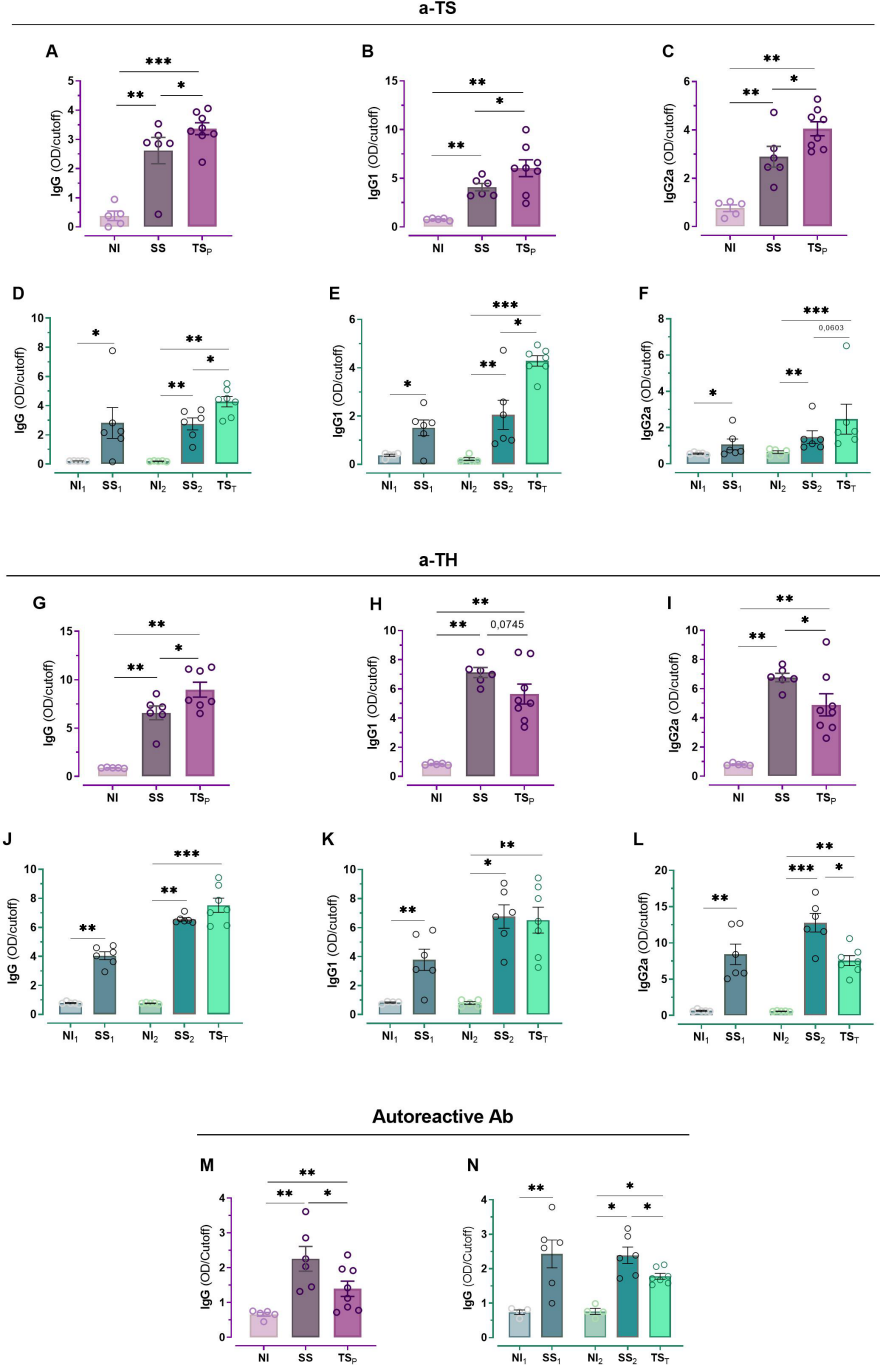
Antibody and auto-reactive antibody responses in the chronic phase of infection allowing prophylactic or therapeutic vaccination. Anti-TS antibodies response (a-TS) were assessed for total IgG **(A, D)**, IgG1 **(B, E)** and IgG2a **(C, F)** under prophylactic (NI = 5, SS = 6, TS_P_=8) and therapeutic (NI_1_ = 5, SS_1_ = 6, NI_2_ = 5, SS_2_ = 6, TS_T_=7) schemes, respectively. Anti-homogenate (a-TH) response was also evaluated for total IgG **(G, J)**, IgG1 **(H, K)**, and IgG2a **(I, L)** under each scheme. IgG auto-reactive antibodies were also determined for prophylactic **(M)** and therapeutic **(N)** schedules. Antibody levels are indicated as OD relative to the cut-off value of the ELISA assay. Statistical significance is indicated as follows: *p < 0.05 and **p < 0.01 and ***p < 0.001.

Compared to infected but unvaccinated SS mice, antibody levels against the vaccine antigen were higher in animals immunized prophylactically (TS_P_), including total IgG, IgG1, and IgG2a levels (**Figures 5A–C**). In the therapeutic schedule, TS-specific antibody levels in unvaccinated mice remained similar between the pre-treatment time point at 54 dpi and 135 dpi (SS_1_ vs SS_2_, IgG1: p= 0,4178, IgG2a: p= 0,2279 and IgG total p= 0,2424.). Total TS-specific IgG and IgG1 levels were significantly higher in the TS_T_ group than in SS_2_ mice (**Figures 5D, E**). For IgG2a, TS-specific antibody levels tend to be higher in vaccinated mice in TS_T_ relation to SS_2_ mice, but they do not reach statistical significance (**Figure 5F**).

As expected, during the chronic stage of infection, all infected mice developed antibodies against parasite total homogenate –TH– (**Figures 5G–L**). Vaccinated TS_P_ and TS_T_ mice showed similar TH-specific total IgG levels compared to their respective unvaccinated SS and SS_2_ groups (**Figures 5G, J**). Remarkably, in both prophylactic and therapeutic treatments, vaccinated mice displayed lower anti-TH-specific IgG2a responses than unvaccinated infected groups (**Figures 5I, L**).

To assess whether the treatments modulated levels of heart auto-reactive antibodies and thereby contributed to reduced tissue damage, plasma from all infected mice was tested by ELISA against a homogenate of healthy BALB/c heart tissue. As expected, NI controls showed no detectable response, whereas unvaccinated infected mice (SS, SS_1_ and SS_2_) exhibited elevated levels of heart auto-reactive antibody. Notably, both vaccination regimens markedly reduced these levels (**Figures 5M, N**).

## Discussion

Many studies have evaluated the effectiveness of prophylactic vaccines in preventing or mitigating tissue damage caused by chronic *T. cruzi* infection (5, 12, 13, 24–26). In addition, many researches have examined the impact of administering therapeutic vaccines during the chronic stage of infection (27–31). Some of these authors have even compared therapeutic and prophylactic vaccines within the same infection model and using the same vaccine formulation (5, 29, 31). However, to our knowledge, this type of analysis has not yet been conducted for nasal vaccines as performed in the present report. A key distinction is that unlike most studies that rely on intraperitoneal challenges, the oral infection model used in our study offers a more realistic representation of parasite-host invasion, more closely mimicking one of the natural infection routes. In 13, we first demonstrated that a nasal prophylactic vaccine using the same formulation as in the present study improves the course of both the acute and chronic stages of the infection. Since most *T. cruzi* infections are diagnosed only at the chronic stage—when parasiticidal treatment is no longer effective—and more than 6 million people are currently living with chronic infection, the development of effective therapies for this stage is critical. Here, we report for the first time a study evaluating the potential of a nasal therapeutic vaccine to prevent chronic complications in a model of oral *T. cruzi* infection, and compare its effects with our previously established prophylactic regimen.

The presence of inflammatory infiltrates and fibrotic lesions are two hallmark features of CCC at the histological level, and they correlate with the clinical severity of heart failure (32–35). These lesions are associated with progressive cardiac dilation and failure, reason why they are considered key contributors to Chagas-related cardiac dysfunction (36). Typically, in the chronic phase of infection of mice, the inflammatory infiltrate is of low to moderate intensity (37, 38). In particular, in female BALB/c mice infected with the Tulahuen strain, we have previously reported the development of moderate inflammation and fibrosis, regardless of the route of infection (13, 21). In the present study, we have reproduced these results and notably, both inflammatory infiltrate and fibrosis were significantly reduced not only in TS_P_, as we have previously described, but also in TS_T_ group.

Electrocardiographic alterations, together with histological analysis, represent a valuable approach to assess cardiac lesions caused by *T. cruzi* infection (38). Although these alterations do not fully replicate human chronic Chagas cardiomyopathy, they serve as a potential indicator of cardiac functional damage. Furthermore, studies conducted in dogs, a model with notable similarities to human pathology, have shown that prophylactic vaccines prevent ECG alterations in infected animals (39). In BALB/c mice specifically infected through diverse routes with Tulahuen strain, we and others have demonstrated that Chronic murine myocarditis results in electrocardiographic alterations (13, 21, 38, 40). In our model, the therapeutic regimen in TS_T_ mice was not effective in preventing QRS and QTc alterations compared to the prophylactic treatment, but the number of affected mice tended to be significantly lower in this group than in the SS group of infected controls, suggesting a functional benefit. These findings align with previous studies also showing that therapeutic vaccines administrated by other routes can prevent electrocardiographic abnormalities and histological damage in mice and dogs (28, 41–43). More recently, *Rhesus macaques* chronically infected and treated with a DNA vaccine during the chronic stage showed improvements in electrocardiographic parameters (44).

The effectiveness of both intranasal vaccination regimens was also evident in their ability to reduce parasite burden. In the case of the prophylactic vaccine, we have already determined in previous works, that the parasite load remains low throughout both the acute and chronic stages of infection (13, 6). Given the favorable immune profile observed previously in these mice during the acute phase, we attribute this control to the immune system’s ability to manage the infection. While not sterilizing, it effectively reconfigured immune function, preventing parasite dissemination and lesion development. Importantly, the intranasal therapeutic vaccine is also capable of lowering parasite burden, even though the parasite had already been established in cardiac tissue before its administration. The presence of parasites in the heart is considered a key determinant for lesion establishment (45, 46), and some authors have reported a correlation between parasite load and lesion severity (33, 47, 48). For this reason, a reduced parasite burden may have helped improve cardiac parameters observed with both vaccination regimens.

In our previous work, the mucosal immune response induced by this vaccine formulation was thoroughly characterized, showing stimulation of IgA secretion at mucosal surfaces, as well as increased proliferation of CD4^+^, CD8^+^, and antigen-specific B lymphocytes in the nasopharynx-associated lymphoid tissue (NALT), cervical lymph nodes (CLN), and spleen. Additionally, it enhanced cytokine profiles in NALT, including IFN-γ, IL-17A, IL-4, IL-5, and IL-21 (13). In the present study, since our goal was to evaluate the therapeutic potential of this formulation once the parasite is chronically established, we focused first on characterizing the immune response in heart tissue—the organ most affected in chronic Chagas cardiomyopathy (CCC)—to understand how the vaccine might influence infection outcomes during the chronic stage. The profile of systemic antibodies was also evaluated, considering their potential involvement in either protective or deleterious effects during chronic infection. Indeed, these results cannot be interpreted in a single way as there is no consensus on the optimal immune response a vaccine should elicit during chronic infection to prevent cardiac damage. Still, comparisons of cytokine profiles in chronic patients with and without cardiomyopathy provide valuable insights into potentially protective immune mechanisms (49), and the same criteria may be true for antibodies (50). In our model, SS groups—which correspond to infected, untreated mice that develop cardiac lesions—showed increased expression levels of IFN-γ and T-bet in heart tissue compared to NI controls. This cytokine pattern resembled systemic assessment of immune mediators analyzed in CCC patients as revised by Koh et al. (51). In fact, systemic and cardiac elevated IFN-γ levels has been strongly linked to increased cardiac damage in both mouse models and human patients (49, 51, 52). Interestingly, when IFN-γ expression was analyzed in heart, the levels were low in TS_P_ and high in TS_T_ in relation to SS groups.

Fibrosis in *T. cruzi* infection has also been linked to TGF-β, often acting alongside IFN-γ (60). The deleterious effect of TGF-β was confirmed in a murine model of *T. cruzi* infection, where neutralization of this cytokine led to a reduction of fibrosis, as well as improved cardiac function (53). Consistent with this finding and our own earlier prophylactic studies (13), TS_P_-vaccinated animals showed lower TGF-β expression, likely indicating reduced fibrotic remodeling, which correlates with the decreased cardiac fibrosis observed in this group. Differently, the therapeutic vaccine did not reduce TGF-β expression. However, in this approach, TS_T_ animals showed higher T-bet expression compared to NI_2_ and also elevated Foxp3 expression compared to SS_2_, possibly reflecting the delayed onset of immune modulation. These differences likely stem from the timing of immune priming: in the prophylactic scheme, early activation during the acute phase may promote infection control and long-term immune equilibrium, characterized by lower fibrotic and pro-inflammatory cytokine levels (54). Conversely, therapeutic vaccination in the chronic stage appears to induce a different profile, where inflammatory response seems to be balanced by Foxp3+ expressing Tregs. Of note, these results may be potentially indicative of a Th1-like phenotype in Foxp3^+^ cells, as previously reported in experimental *T. cruzi* models (61).

Myocardial IL-17 expression was markedly increased in the myocardium of both TS_P_- and TS_T_-vaccinated animals compared to their respective SS and SS_2_ controls. Higher IL-17 levels have been associated with decreased IL-12 expression and also with the asymptomatic forms of the disease, suggesting a protective role (51, 55). Recent findings have shown a strong association between preserved cardiac function and IL-17–producing T cells in patients, highlighting this cytokine as a potential therapeutic target (56). Specifically, TS_P_ animals exhibited reduced T-bet and enhanced IL-17 expression in cardiac tissue, indicative of a cytokine environment linked to reduced damage. Similarly, IL-17 was markedly upregulated in TS_T_ mice. This suggests a central role for this cytokine—either directly or via regulatory T cells—in mediating vaccine-induced cardiac protection. This is consistent with findings that Th17 cells contribute to both intracellular and extracellular control of *T. cruzi* (57), and their protective potential has been suggested to surpass that of Th1 cells (58). In parallel, an increase in FOXP3 was detected in the TS_T_ group, which would suggest that, together with IL-17, a regulatory effect might be occurring to balance the elevated IFN-γ levels that persist in this group. Nonetheless, this increase could not be correlated with IL-10 levels in the same group.

Taken together, these data indicate that while both vaccination strategies confer cardiac protection, they rely on different immune mechanisms. Cytokine signaling pathways, particularly IL-17-related ones, appear to play a particularly prominent role in mediating efficacy. Understanding how cytokine networks shift after immunization is therefore crucial to uncovering the protective mechanisms in CCC.

An aspect that remains underexplored in studies on therapeutic vaccines for Chagas disease is the characterization of the antibody profile elicited following treatment. Our findings show that the prophylactic vaccine induces a broad humoral response, boosting the production of both antibody subtypes against the vaccine antigen. By contrast, therapeutic vaccination primarily promotes a predominant increase in IgG1 levels, with only a modest rise in IgG2a. These results suggest that while both strategies successfully trigger antigen-specific humoral responses, they do so with distinct subtype proportions.

Notably, the antibody response triggered by the nasal prophylactic vaccine persists into the chronic phase of infection, indicating long-term immunogenicity. Moreover, both vaccination strategies were linked to reduced antibody levels against total parasite antigens, suggesting a shift in humoral focus—from wide parasite recognition toward the specific vaccine antigen. This pattern—elevated vaccine-specific antibodies and reduced reactivity to parasite homogenate—mirrors our previous findings using a subcutaneous trans-sialidase (TS)-based therapeutic vaccine (21) and may contribute to the observed protective effect, considering that certain parasite-directed antibodies can be detrimental to immune efficacy. In chronic Chagas disease, two major classes of antibodies are typically present (59). The first includes lytic antibodies that target parasite surface proteins and mediate protection via complement activation and antibody-dependent cellular cytotoxicity. The second class comprises conventional serological antibodies, which, although useful for diagnosis, do not contribute to parasite control.

Building on these findings, the antibody profile induced by our vaccines, marked by a shift toward a more targeted and potentially functional humoral response, may help to attenuate disease progression in chronic infection. Our current analysis shows a notable increase in protective antibodies against TS, along with a decrease in other *T. cruzi*-specific antibodies, many of which may be non-protective or even pathogenic. This is especially relevant in light of two key findings. First, TS is a surface molecule, and we previously demonstrated that antibodies induced by the prophylactic nasal vaccination reduce parasite invasion *in vitro* into eukaryotic cells and enhance the survival of passively immunized mice challenged with *T. cruzi* (6). Second, we have reported antibody-mediated cytotoxicity using a recombinant BCG vaccine expressing TS (11).

Certain harmful antibodies described in *T. cruzi* infection have been linked to autoimmune events (50). Although the precise role of these autoantibodies in the clinical progression of Chagas disease remains unclear, their potential involvement in pathogenesis has been supported by passive transfer studies showing that they can reproduce cardiac damage in animal models (50). In our experiments, both therapeutic and prophylactic vaccination led to a reduction in auto-reactive antibodies levels against cardiac tissue. This reduction—along with the favorable cytokine profile, lower parasite load and specific antibodies elicited by the vaccines—may collectively contribute to the cardioprotective effects seen with both vaccination strategies. Since molecular mimicry has been proposed as a mechanism behind the generation of auto-reactive antibodies (50), it is plausible that the antibodies reduced by the intervention are linked to ongoing lower parasite persistence. Their decline may reflect a decreased parasite burden during the chronic phase, which could in turn limit the sustained autoimmune events.

**Figure 6:**
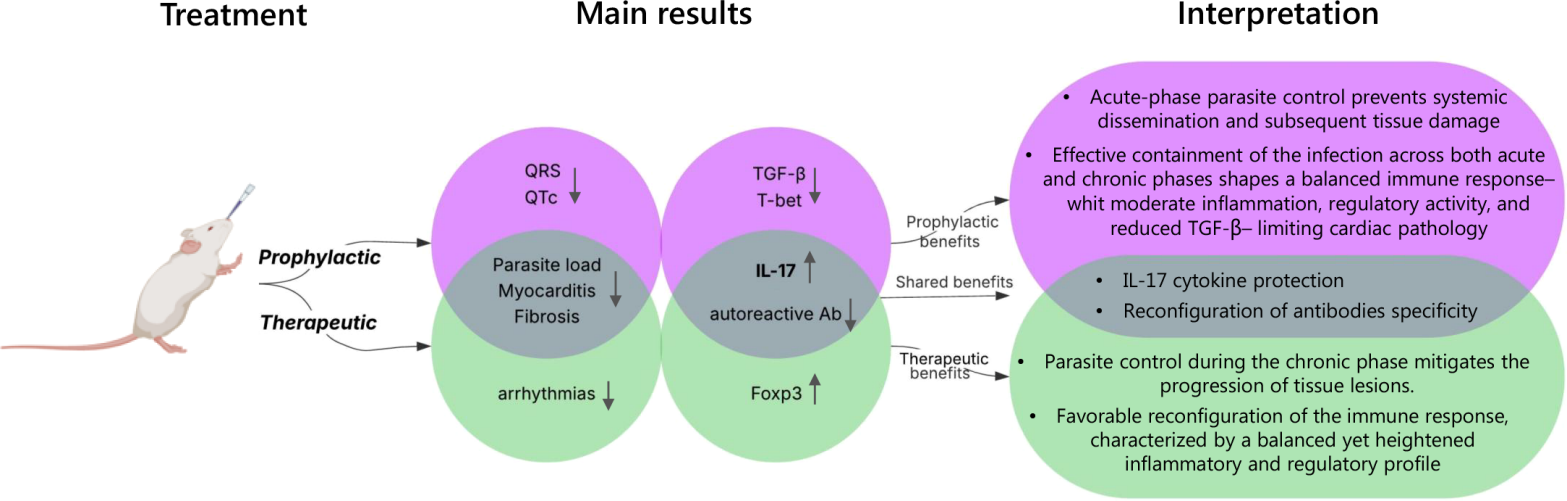
Summary of the main results and their possible interpretation for both the prophylactic and therapeutic schemes.

In summary, our results suggest that the TS + c-di-AMP vaccine can prevent chronic tissue damage when administered intranasally, both prophylactically and therapeutically, with the prophylactic strategy providing superior protection. As illustrated and interpreted in **Figure 6**, this enhanced efficacy may stem, at least in part, from early mitigation of tissue damage during the acute phase of infection, along with a favorable immune profile previously described in this model (13). In contrast, therapeutic vaccination occurs after acute-phase injury has already taken place—as observed at 54 days post-infection—and must act during the chronic phase to repair or limit further damage. Despite this, the therapeutic approach still led to improvements in functional parameters, though these remained below pre-infection levels. These functional gains were accompanied by a marked reduction in inflammatory infiltrates and fibrosis. These findings are consistent with the observations reported by other authors, who documented attenuated pathology using a single formulation as prophylactic or therapeutic vaccination but delivered in a systemic way (5, 29, 31). We foresee additional studies with larger cohorts to more comprehensively characterize the immune responses induced by our nasal vaccination strategies and to elucidate in greater detail the underlying protective mechanisms involved. In this context, both innate and adaptive immune cells represent key components that should be systematically analyzed. Furthermore, although the oral infection model employed in this study enhances the translational relevance of the work by mimicking a major natural transmission route increasingly recognized in humans, it will also be important in the future to evaluate the efficacy of this strategy against other relevant infection routes. Vector-borne transmission remains the principal route globally, while congenital and transfusional transmissions continue to contribute substantially to the overall disease burden. Bearing in mind that distinct routes of infection may shape host–parasite interactions and immune responses in different ways, thereby potentially modulating mucosal vaccine efficacy, the assessment of cross-protection across multiple infection routes constitutes an essential objective for future research.

## Data Availability

The raw data supporting the conclusions of this article will be made available by the authors, without undue reservation.
